# Myeloid cell reprogramming alleviates immunosuppression and promotes clearance of metastatic lesions

**DOI:** 10.3389/fonc.2022.1039993

**Published:** 2022-11-21

**Authors:** Ravi M. Raghani, Jeffrey A. Ma, Yining Zhang, Sophia M. Orbach, Jing Wang, Mina Zeinali, Sunitha Nagrath, Sandeep Kakade, Qichen Xu, Joseph R. Podojil, Tushar Murthy, Adam Elhofy, Jacqueline S. Jeruss, Lonnie D. Shea

**Affiliations:** ^1^ Department of Biomedical Engineering, University of Michigan, Ann Arbor, MI, United States; ^2^ Department of Chemical Engineering, University of Michigan, Ann Arbor, MI, United States; ^3^ COUR Pharmaceuticals Development Co, Inc, Northbrook, IL, United States; ^4^ Department of Microbiology-Immunology, Feinberg School of Medicine, Northwestern University, Chicago, IL, United States; ^5^ Department of Surgery, University of Michigan, Ann Arbor, MI, United States; ^6^ Department of Pathology, University of Michigan, Ann Arbor, MI, United States

**Keywords:** metastasis, polymeric nanoparticles, immune cell reprogramming, cancer immunotherapy, triple negative breast cancer

## Abstract

Suppressive myeloid cells, including monocyte and neutrophil populations, play a vital role in the metastatic cascade and can inhibit the anti-tumor function of cytotoxic T-cells. Cargo-free polymeric nanoparticles (NPs) have been shown to modulate innate immune cell responses in multiple pathologies of aberrant inflammation. Here, we test the hypothesis that the intravenous administration of drug-free NPs in the 4T1 murine model of metastatic triple-negative breast cancer can reduce metastatic colonization of the lungs, the primary metastatic site, by targeting the pro-tumor immune cell mediators of metastatic progression. *In vivo* studies demonstrated that NP administration reprograms the immune milieu of the lungs and reduces pulmonary metastases. Single-cell RNA sequencing of the lungs revealed that intravenous NP administration alters myeloid cell phenotype and function, skewing populations toward inflammatory, anti-tumor phenotypes and away from pro-tumor phenotypes. Monocytes, neutrophils, and dendritic cells in the lungs of NP-treated mice upregulate gene pathways associated with IFN signaling, TNF signaling, and antigen presentation. In a T-cell deficient model, NP administration failed to abrogate pulmonary metastases, implicating the vital role of T-cells in the NP-mediated reduction of metastases. NPs delivered as an adjuvant therapy, following surgical resection of the primary tumor, led to clearance of established pulmonary metastases in all treated mice. Collectively, these results demonstrate that the *in vivo* administration of cargo-free NPs reprograms myeloid cell responses at the lungs and promotes the clearance of pulmonary metastases in a method of action dependent on functional T-cells.

## Introduction

Globally, breast cancer has the highest rates of incidence and mortality among women (1). Among breast cancer subtypes, triple negative breast cancer (TNBC) is the most difficult to manage, and few therapies exist to effectively treat high risk TNBC. Since TNBC is one of the most immunogenic breast cancer subtypes, immunomodulatory therapies have the potential to significantly improve disease outcomes, and clinicians have turned to these therapies to manage disease (2, 3). Since 2020, the T-cell-stimulating checkpoint inhibitor pembrolizumab has been approved for metastatic, locally advanced, and early TNBCs (4, 5). However, while robust results have been observed in TNBC patients responsive to pembrolizumab, the majority are resistant to therapy, and a significant proportion of TNBC patients receiving this immunotherapy experience grade 3 or higher adverse events. The high proportion of therapy-resistant patients and inability of some patients to tolerate pembrolizumab motivates the development of novel immunotherapies to improve disease outcomes.

Cancer immunotherapies present the opportunity to stimulate the immune system to mount a potent anti-tumor response. Whereas the immune system of a healthy individual utilizes both innate and adaptive immune surveillance mechanisms to eliminate neoplastic cells, cancer-induced immune dysregulation suppresses many of these mechanisms and drives the generation of aberrant, pro-tumor immune cells (6, 7). Tumor-secreted factors induce the formation of immature, immunosuppressive myeloid cells, largely neutrophils and monocytes, which have been shown to facilitate the formation of a pre-metastatic niche, recruit disseminated tumor cells to the metastatic niche, and suppress adaptive immunity to promote the progression of metastatic disease (8–10). Immunosuppressive neutrophils secrete arginase-1 and reactive oxygen species, such as nitric oxide and hydrogen peroxide, which can inhibit T-cell proliferation and function (11–13). Similarly, monocyte-derived tumor-associated macrophages suppress T-cell activation through their expression of PD-L1 and HIF-1α (14, 15). The immunosuppressive and tumor-supportive function of myeloid cells has been implicated in resistance to cancer therapies, including checkpoint blockade (16).

Myeloid cells in the metastatic niche directly support tumor cell colonization and correlate with poor clinical outcomes, and thus serve as highly compelling targets for the next generation of immunotherapies (17). Approaches to target myeloid cells include the depletion of these populations with anti-Gr-1 or clodronate liposomes, and the inhibition of signaling molecules, such as CCL2 or G-CSF, which drive myeloid cell recruitment to metastatic sites (18). In murine models, depletion of neutrophil and monocyte populations enhances anti-tumor immunity and inhibits metastatic seeding (19, 20). Likewise, targeted inhibition of CCL2 can block recruitment of tumor-supportive monocytes to the lung and inhibit metastasis (10). While these immunotherapies have shown some early promise, non-specific cell depletion or the systemic blocking of signaling pathways has demonstrated poor results in clinical trials due to its unintended inhibition of tumor-suppressive myeloid cell populations, in addition to pro-tumor populations (21). Inflammatory, anti-tumor subsets of neutrophils and macrophages can stimulate T-cell cytotoxicity and play an important role in controlling tumor growth (22, 23). Novel immunotherapies that skew immunosuppressive myeloid cells toward anti-tumor phenotypes, as opposed to broadly depleting cell populations, have the potential to more effectively treat metastasis.

Immunomodulatory nanoparticles (NPs) are being developed to target pathological inflammation in cancer and other diseases. Traditionally, nanoparticles have been used to deliver chemotherapies and other pharmaceuticals to tumors (24, 25). Recently, cargo-free polymer nanoparticles have been shown to modulate immune cell behavior in a tunable manner dependent on their physiochemical properties (26, 27). Upon intravenous delivery, nanoparticles are taken up by myeloid cells, such as macrophages, neutrophils, and dendritic cells (28). Nanoparticle administration alters myeloid cell phenotype and redirects myeloid cell trafficking away from sites of inflammation toward the spleen (29). The efficacy of drug-free nanoparticles to mitigate pathological inflammation by modulating myeloid cells has been reported in models of traumatic injury and autoimmune disease, and has also been shown to inhibit primary tumor growth in a murine model of breast cancer (30–32).

In this report, we investigate the hypothesis that intravenous administration of cargo-free poly(lactide-co-glycolide) (PLG) nanoparticles can reduce metastatic colonization of the lungs by targeting the immunosuppressive, myeloid cell mediators of metastatic progression. In an orthotopic murine model of metastatic triple negative breast cancer (4T1), metastatic tumor cells seed and colonize the lungs, orchestrated in part by the recruitment of aberrant myeloid cells to the lungs (33). The impact of nanoparticles on circulating tumor cells, pulmonary metastasis, and the lung microenvironment are analyzed *in vivo*. Gene expression changes among distinct leukocyte populations within the metastatic niche, resulting from nanoparticle administration, are investigated with single-cell RNA sequencing. Finally, the efficacy of nanoparticle delivery on tumor cell clearance is studied in a T-cell-deficient murine model and primary tumor resection model. These cargo-free nanoparticles provide a novel platform for modulating cancer-associated myeloid cells and enhancing anti-tumor T-cell surveillance, and as such, demonstrate great potential as a neoadjuvant and adjuvant therapy for treating advanced cancers.

## Materials and methods

### Nanoparticle fabrication

COUR Pharmaceuticals Development Co, Inc. manufactured ONP-302, the PLG nanoparticles (NPs) used in these studies. Briefly, NPs were made from PLG (Lactel^®^, Durect Corporation) using a double emulsion technique. A water-in-oil emulsion containing a proprietary mixture of PLG and surfactants was prepared. Solvents were removed by evaporation, yielding negatively charged NPs which were then washed, filtered, and concentrated by tangential flow filtration. ONP-302 NPs were then formulated with buffering agents and cryoprotectants, and lyophilized. The physiochemical properties of nanoparticles, shown in **Figure S1**, were characterized by dynamic light scattering (DLS) and scanning electron microscopy (SEM).a

### Tumor cell culture and animal inoculations

4T1-luc2-tdTomato murine triple negative breast cancer cells (PerkinElmer) were cultured in RPMI 1640 Medium (Gibco) containing 10% fetal bovine serum (FBS, Avantor) for 5 days (37°C, 5% CO2) prior to orthotopic inoculation. Tumor cells were enzymatically lifted from the tissue culture flask with trypsin (Gibco) for 10 minutes at 37°C and resuspended in culture medium. Cells were centrifuged at 500xg for 5 minutes and resuspended in Dulbecco′s phosphate buffered saline (DPBS) (Gibco) at a concentration of 2E6 cells/mL. The tumor cell line was previously confirmed to be pathogen free and authenticated by short tandem repeat DNA analysis and compared to the ATCC STR profile database (DDC Medical). Orthotopic inoculations were performed by injecting 50 µL of the cell suspension, containing 1E5 4T1 tumor cells, to the fourth right mammary fat pad of 12-week-old female BALB/c mice (Jackson Laboratory strain #000651). In addition to the immunocompetent BALB/c strain, RAG1-KO (Jackson Laboratory strain #003145) mice, which have a BALB/c background, were used as they are homozygous for the Rag1^tm1Mom^ mutation and thus do not produce mature T-cells. All animal studies were reviewed and approved by University of Michigan Institutional Animal Care & Use Committee.

### Nanoparticle administration

Nanoparticles were intravenously injected into the lateral tail veins of mice. NPs were resuspended in a mixture of sterile water for injection (Intermountain Life Sciences) and sterile DPBS. NPs were first resuspended in water at 40 mg/mL and diluted with DPBS to 10 mg/mL for a final ratio of 1 parts water to 3 parts DPBS. NPs were administered at a dose of 1 mg in 100 μL of solution every 3 days. Control mice received 100 μL of DPBS. NPs were injected starting 1, 2, or 4 days after tumor inoculation for primary tumor growth studies. In tumor resection studies, the neoadjuvant group received NPs starting 1 day after inoculation, and additional doses every 3 days until tumor resection at 11 days, for a total of 4 doses. Mice in the adjuvant group did not receive NPs prior to resection, and initiated dosing 1 day after resection with NPs every 3 days until the endpoint at 42 days after resection. For all other studies, nanoparticles were administered starting 1 day after tumor inoculation and continued every 3 days until the study endpoint.

### Tumor volume measurements

Tumor size was recorded using standard electronic calipers (VWR) while mice were anesthetized with 2% v/v isoflurane. Primary tumor volume was calculated (V = 0.5 x L x W2, L: length of longest dimension of the tumor, W: width perpendicular to the longest tumor dimension) as previously described (32).

### *Ex vivo* bioluminescent imaging

Metastatic burden at the lungs was measured with bioluminescent imaging of explanted whole organs at terminal endpoint using an IVIS Lumina LTE imaging system (Caliper Life Science). Tissues were incubated in DPBS containing 630 μM d-luciferin (Caliper) at 37°C for 10 minutes and subsequently imaged with the IVIS. The bioluminescent imaging of luciferase-expressing 4T1 cells were calculated by the Living Image Software (Caliper Life Sciences) and visualized as flux (photons/sec) from the tissues.

### Circulating tumor cell quantification and association with immune cells

Circulating tumor cells (CTCs) were isolated and quantified using the Labyrinth microfluidic platform as previously described (34). CTCs are separated from leukocytes by size, as white blood cells are smaller than 4T1 cells. Briefly, blood samples were collected from mice treated with nanoparticles 14 days after tumor inoculation, from tumor-bearing mice injected with saline at 14 days after inoculation, and from naive mice without tumors. Blood from two mice were collected in EDTA tubes and pooled per sample. Prior to CTC enrichment, red blood cells were removed using Ficoll-Paque™ PLUS Media. The plasma and buffy coat layers were collected and diluted with DPBS (1:7.5) and processed through the Labyrinth. After CTC isolation, samples were taken from outlet 2 and processed using a Thermo Scientific Cytospin Cytocentrifuge for CTC quantification. A portion of sample was added to each cytospin funnel and centrifuged at a speed of 800 rpm for 10 minutes. Samples were fixed with 4% PFA, permeabilized with 0.2% Triton X-100, and blocked with 3% bovine serum albumin. Samples were then stained with anti-mouse CD45 antibody and DAPI. Two slides were imaged from each outlet for each sample for technical replicates. Representative images of CTCs and leukocytes are shown in **Figure S2B**.

### Single-cell RNA sequencing

Saline- or NP-treated lungs were extracted and pooled from five mice 14 days post-tumor inoculation. The tissues were minced with scalpel blades, enzymatically digested with liberase TM (Roche), and processed through a cell strainer. Cell suspensions underwent erythrocyte lysis with ACK buffer (Gibco) for 5 minutes. Library preparation of the resulting single cell suspension was conducted with the 10x Chromium platform. Samples were sequenced on the NovaSeq 6000 at the University of Michigan Advanced Genomics Core at an average depth of 50,000 reads per cell. Raw reads were mapped by CellRanger to output count matrices with subsequent clustering and differential gene expression performed using the Seurat (v2) pipeline (35). Cells were identified as having 500 to 5000 genes and < 7.5% mitochondrial genes. Cluster cell types were classified using the markers shown in **Table S1** and **Figures S3A** **S4B**. The clusters defined as neutrophils, monocytes, and dendritic cells were further analyzed to identify specific subsets using the gene expression outlined in **Table S2** and **Figures S3B–D**.

### Gene set enrichment analysis

Within the neutrophils, monocytes, and dendritic cells, differentially expressed genes between the NP-treated and saline-treated cells were identified using Seurat. The resulting gene lists were converted to human orthologs with biomaRt. Gene fold-changes were used as the ranking variable to assemble pre-ranked gene lists. GSEA (pre-ranked gene list) was used to identify enriched pathways sampling from hallmark, KEGG, REACTOME, and gene ontology (GO) databases from the Molecular Signatures Database (MSigDB) (36). The top 10 pathways up-regulated in the NP-treated samples were reported for each cell type with redundant pathways removed by leading edge analysis.

### Flow cytometry, magnetic activated cell sorting, and enzyme-linked immunosorbent assay

Lungs and whole blood of tumor-bearing mice were isolated at 14 days-post tumor inoculation. Lungs were mechanically and enzymatically digested to form single cell suspensions as described above. Following erythrocyte lysis, lung and blood cell suspensions were washed in DPBS supplemented with 2 mM EDTA and 0.5% bovine serum albumin, and centrifuged at 500xg for 5 minutes. For flow cytometric analysis, blood cells were treated with anti-CD16/32 (1:100, clone 93, Biolegend) to block nonspecific staining and samples stained with antibodies against Alexa Fluor 700 anti-CD45 (1:100, clone 30-F11, Biolegend), Brilliant Violet 510 anti-CD11b (1:25, clone M1/70, Biolegend), Brilliant Violet 711 anti-Ly6G (1:20, clone 1A8, Biolegend), and FITC anti-Ly6C (1:100, clone HK1.4, Biolegend), as well as with DAPI (Biolegend) for viability, and analyzed on the BioRad ZE5 Cell Analyzer. Data analysis was performed using FlowJo (BD). To analyze cytokine and chemokine secretion, cells from the blood and lungs were labeled with magnetic microparticle-conjugated antibodies against CD45 (Miltenyi Biotec) and sorted. The CD45-positive fraction, representing immune cells, and CD45-negative fraction, consisting of non-immune cells, were resuspended at 1E6 cells/mL in media and cultured in a 96-well plate for 16-18 hours at 2.5E5 cells/well. Following incubation, cells were centrifuged at 1000xg for 5 min. Supernatant was collected and frozen at -80°C. The levels of IL-1β, IL-4, IL-6, IL-13, IFNγ, TNFα, TGFβ, CCL2, CCL3, CCL4, and CXCL12 in supernatant were quantified *via* ELISA performed by the Cancer Center Immunology Core at the University of Michigan.

### Statistical analysis

Two-tailed unpaired t-tests assuming unequal variance were performed for single comparisons between two conditions. Significant differentially expressed genes in the single-cell RNA sequencing analysis was determined with the Wilcoxon rank-sum test using default Seurat settings. The significance of GSEA pathway scores were determined with Student’s t-tests using the Bonferroni multiple hypothesis correction (α = 0.01). Prism 9 (GraphPad), Excel (Microsoft), and R were used for performing statistical analyses, with p < 0.05 considered to be statistically significant. Error bars on plotted data are calculated as standard error mean (SEM).

## Results

### Nanoparticle administration reduces tumor growth and metastatic dissemination

**Figure 1:**
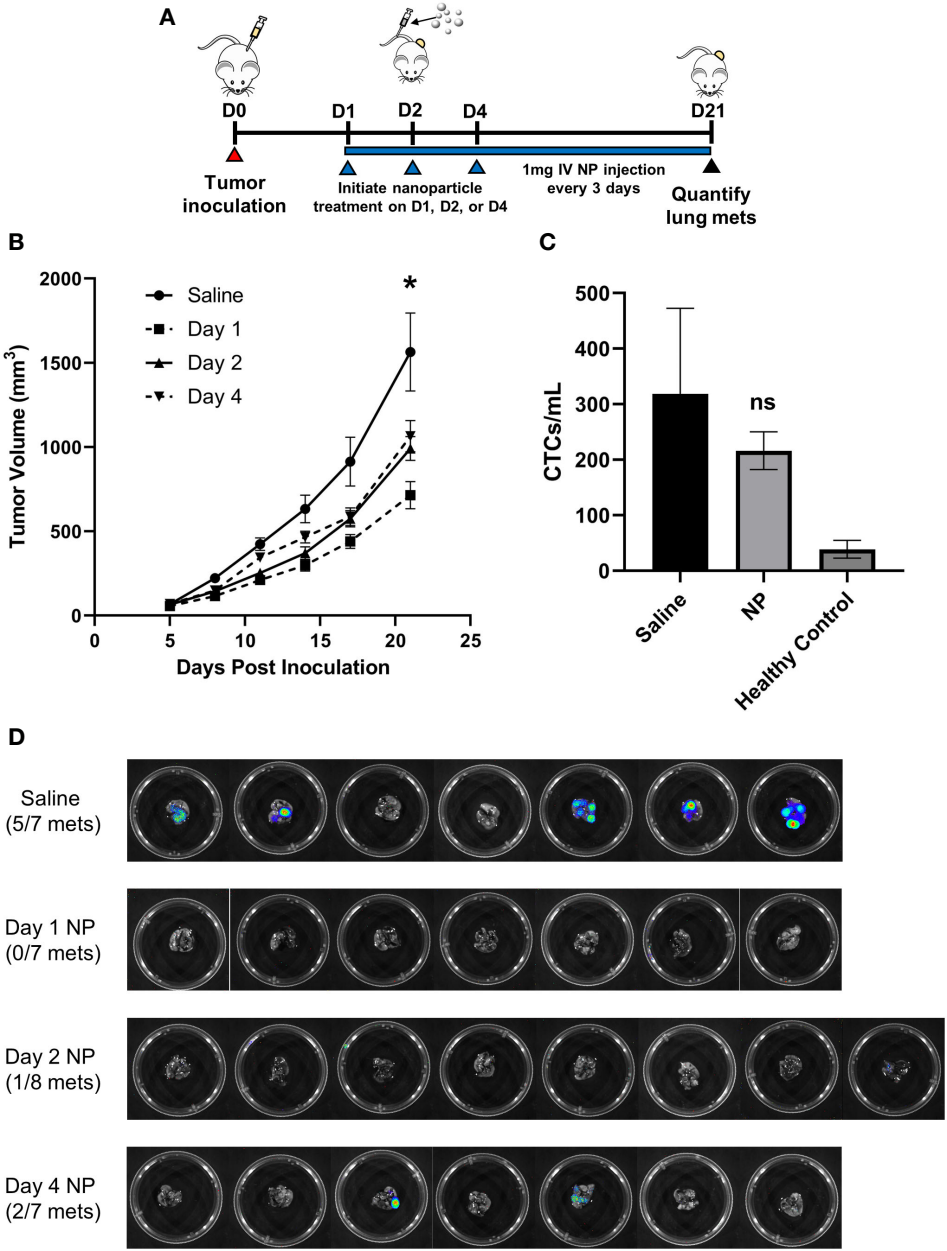
*In vivo* administration of cargo-free NPs inhibits 4T1 tumor growth and reduces metastatic colonization of the lungs. **(A)** BALB/c mice (n=7-8 per group) were inoculated with 4T1 tumor cells and intravenously administered NPs every 3 days starting on D1, D2, or D4 post-tumor inoculation, or saline as the control. **(B)** Longitudinal PT volumes. **(C)** Total CTCs in the peripheral blood of 4T1 tumor-bearing mice (n=6) receiving NPs, normalized to blood volume, did not significantly differ from mice receiving saline (n=6, p=0.51). **(D)** Lung metastases in 4T1 tumor-bearing mice (n=7-8 per group) were quantified with bioluminescent imaging on D21 post-tumor inoculation. Two-tailed unpaired t-tests assuming unequal variance were performed for single comparisons between two conditions. Bars show mean ± SEM. * p ≤ 0.05 compared to Day 1 NP, ns p > 0.05 compared to Saline.

The efficacy of PLG nanoparticles (NPs) to inhibit tumor progression was investigated in the 4T1 model of murine metastatic TNBC, in which neutrophils and macrophages are known to promote tumor development (37). We first tested the hypothesis that NP-mediated immunomodulation would disrupt the growth of primary tumors. BALB/c mice were orthotopically inoculated with 4T1 cells and intravenously injected with NPs (1 mg/mouse) starting 1, 2, or 4 days after tumor inoculation to study how NPs disrupt tumor growth and formation (**Figure 1A**). We hypothesize that 4T1 tumor cells begin engrafting 1 day after inoculation, complete engraftment by 2 days, and will have established into palpable tumors by 4 days post-inoculation. Twenty-one days after inoculation, mice receiving NPs starting on day 1, 2, or 4 exhibited a 54% (p<0.05), 37% (p<0.05), and 32% (p=0.068) reduction in tumor size, respectively, relative to saline-treated controls (**Figure 1B**). Furthermore, mice treated with NPs starting 1 day after inoculation had significantly smaller tumor volumes at day 21 compared to mice treated starting 2 days (28% reduction, p<0.05) or 4 days (33%, p<0.05) after inoculation. As such, we concluded that early administration of NPs effectively disrupted tumor growth.

Next, we examined NP efficacy at inhibiting tumor cell dissemination and metastasis. As orthotopic 4T1 tumors are known to spontaneously metastasize to the lungs, we investigated metastatic colonization of the lungs *via* bioluminescent imaging of luciferase-expressing 4T1 cells. NP administration starting 1 day after inoculation completely blocked formation of pulmonary metastases, while 5 of 7 mice receiving saline developed metastases (p<0.01, **Figure 1D**). In comparison, 1 of 8 mice treated with NPs starting 2 days after inoculation and 2 of 7 mice treated starting 4 days after inoculation developed metastases (p<0.05 and p=0.13, respectively, compared to saline). All subsequent studies were performed initiating NP administration 1 day post-tumor inoculation, motivated by the robust efficacy at this time point. To investigate the mechanism of action by which NP administration inhibits metastasis, we started by evaluating its effect on circulating tumor cells (CTCs) in the blood. Neither the quantity of CTCs per mL of blood (p=0.51, **Figure 1C**) nor the ratio of CTCs to CD45+ immune cells (p=0.79, **Figure S2A**) significantly differed between 4T1 tumor-bearing mice treated with NPs or saline. These findings demonstrate that NP administration significantly reduces the formation of metastatic lesions *in vivo* independently of CTC dissemination.

### Immune cell composition is altered at lung microenvironment with nanoparticle administration

**Figure 2:**
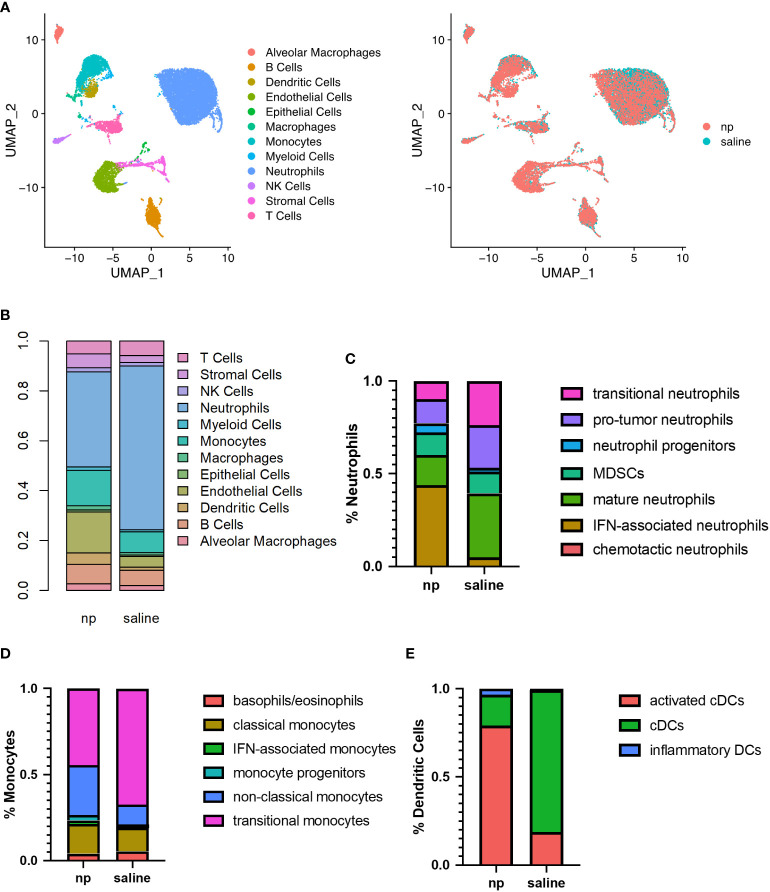
Remodeling of immune milieu at lungs due to NP administration *in vivo*. The lungs of 4T1-bearing mice receiving NPs or saline (n=4 per group) analyzed by single cell RNA sequencing. **(A)** UMAP plot of clustering of single cells by identified cell populations (left) or by treatment condition (NP vs saline, right). **(B)** Histogram of cell populations at the lungs as percentage of total cells. Proportion of **(C)** neutrophil, **(D)** monocyte, and **(E)** dendritic cell subsets at the lungs of mice receiving NPs or saline.

NPs are primarily phagocytosed by myeloid cells, which mediate the development of the metastatic niche, and as such, we investigated the influence of NPs on the composition and phenotype of the pulmonary metastatic niche (21). We hypothesized that NPs reprogram the metastatic microenvironment and alter the behavior of pro-tumor myeloid cells, including neutrophil and monocyte populations (32). We performed single-cell RNA sequencing on cells isolated from the lungs of tumor-bearing mice receiving either saline or NPs to characterize how immune populations at the metastatic niche are altered with NP administration (**Figure 2A**). In control mice, neutrophils made up 65.5% of all cells in the lung, followed by endothelial cells (10.6%), monocytes (7.7%), and B cells (3.3%) (**Figure 2B**). Lungs from NP-treated mice had a substantially lower proportion of neutrophils (38.1%), and an increased proportion of monocytes (13.8%), endothelial cells (11.4%), and B cells (11.3%). T-cells (6.7%), stromal cells (5.2%), and dendritic cells (4.6%) also increased in NP-treated mice in comparison to saline-treated mice (2.4%, 2.7%, and 1.2%, respectively), suggesting that nanoparticle administration resulted in a lower accumulation of neutrophils and increased proportions of other cell populations.

Neutrophils, monocytes, and dendritic cells demonstrated significant changes in proportion and gene expression in response to NP administration. As such, these myeloid cells, which are capable of NP internalization, were sub-clustered to identify differences in immune cell phenotypes resulting from NP administration. In neutrophils, NP administration was associated with a decrease in pro-tumor subpopulations and a large increase in the proportion of inflammatory neutrophils expressing genes associated with interferon signaling (**Figures 2C**, **S3B**). A 9-fold increase in IFN-associated neutrophils and a 2-fold increase in the proportion of neutrophil progenitors was observed in NP-treated lungs, compared to the saline control. NP administration also resulted in a 2-fold decrease in the proportion of neutrophils identified as pro-tumor and mature, and a 2.5-fold decrease in the proportion of neutrophils identified as transitional. Monocytes similarly demonstrated a 1.5-fold decrease in transitional monocytes and a 2.5-fold increase in the proportion of anti-tumor non-classical monocytes, in the lungs of NP-treated mice (**Figure 2D**). A 2.5-fold increase in cells identified as IFN-associated monocytes with NP administration was also observed, but these made up a small proportion of total monocytes (2.2% in NP-treated mice). Dendritic cells (DCs) shifted from largely immature classical DCs to mature activated DCs with NP administration, exhibiting a 4.5-fold decrease in the proportion of classical DCs and a corresponding 4-fold increase in the proportion of mature activated DCs expressing genes associated with antigen presentation (**Figure 2E**). Similar to neutrophils and monocytes, a population of inflammatory DCs was found primarily in NP-treated mice that strongly expressed genes associated with DC maturation and response to pathogens.

Nanoparticle administration also altered gene expression in T cells at the lung (**Figure S4**). T cells were primarily identified as naïve CD4+ T cells, naïve CD8+ T cells, and Tregs (55.2%, 18.4%, and 12%, respectively, in saline-treated mice). Nanoparticle administration resulted in 1.2-fold and 1.1-fold decreases in the proportions of naïve CD4+ and naïve CD8+ T cells, and corresponding 1.7-fold and 1.5-fold increases in the proportion of Th1/Th2 CD4+ T cells and cytotoxic CD8+ T cells, respectively (**Figure S4A**). Collectively, these data demonstrate that NP administration reprograms the lung microenvironment by decreasing neutrophil accumulation and promoting activated, anti-tumor phenotypes among myeloid cell and lymphocyte populations.

### Nanoparticle delivery results in upregulation of inflammatory pathway-associated genes among myeloid cell populations at the lungs

**Figure 3:**
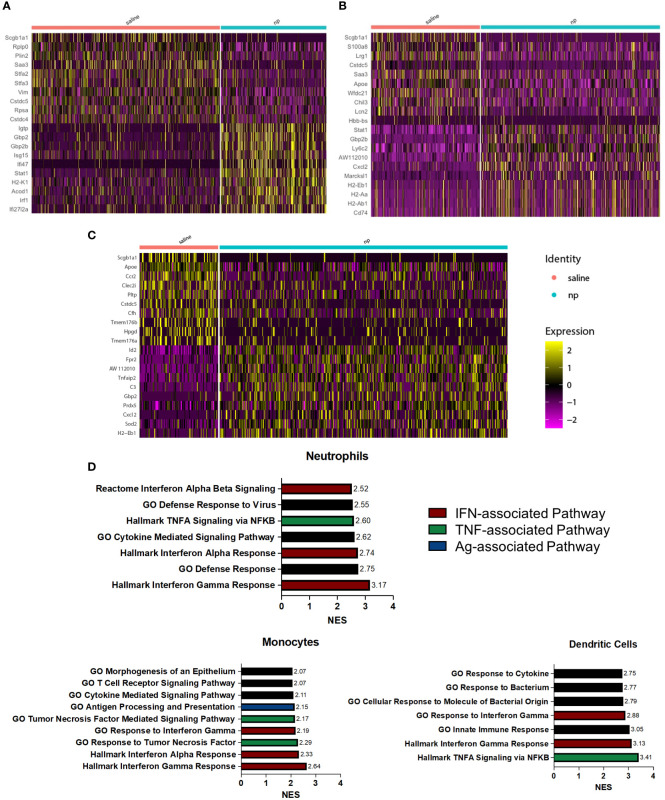
NP administration induces inflammatory, anti-tumor phenotype among pulmonary myeloid cells *in vivo*. Heatmap of differentially expressed genes between **(A)** neutrophils, **(B)** monocytes, and **(C)** dendritic cells at the lungs of 4T1-bearing mice receiving NPs or saline. **(D)** Upregulated pathways among neutrophils, monocytes, and dendritic cells from tumor-bearing mice receiving NPs. Normalized enrichment score (NES) calculated with gene set enrichment analysis.

NP administration resulted in significant changes in gene expression among neutrophils (**Figure 3A**), monocytes (**Figure 3B**), and dendritic cells (**Figure 3C**). In neutrophils, genes associated with inflammation and interferon gamma signaling (*Igtp, Gbp2, Isg15, Ifi47, Stat1*, *Irf1*) were highly upregulated compared to neutrophils in control mice. Upregulation of *Stat1* and *Gbp2b* was also observed in monocytes, along with genes associated with migration (*Cxcl2, Marcksl1*) and antigen presentation (*H2-Eb1, H2-Aa, H2-Ab1, Cd74*). In dendritic cells of NP treated mice, genes associated with inflammation and DC maturation (*Id2, Fpr2, Tnfaip2, Gbp2, Sod2, Cxcl2*) were significantly upregulated compared to the saline control. Gene set enrichment analysis (GSEA) was performed to investigate the functional effects of gene expression changes among these identified populations. All myeloid cell populations saw significant upregulation of pathways associated with IFN and TNF signaling in the NP-treated lungs (**Figure 3D**). The Hallmark Interferon Gamma Response was the top upregulated pathway in neutrophils and monocytes, and the second-most upregulated pathway in DCs. The Hallmark Interferon Alpha Response pathway was also highly upregulated in these cells as the third-most and second-most upregulated pathway in neutrophils and monocytes, respectively. TNFα signaling, as identified in GSEA by both the Hallmark TNFA signaling *via* NFκB and GO Response to Tumor Necrosis Factor gene sets, was also shown to be highly upregulated in neutrophils, monocytes, and DCs. These pathways were the fifth-most, third-most, and top upregulated pathways respectively. IFN and TNF signaling are associated with immune activation and anti-tumor responses in myeloid cells, suggesting that these cells promote adaptive immune responses in NP-treated mice (38–40). Together, these data indicate that nanoparticle administration skews myeloid cells in the lung towards more inflammatory, anti-tumor phenotypes.

### Administration of nanoparticles alters the secretome of immune cells

**Figure 4:**
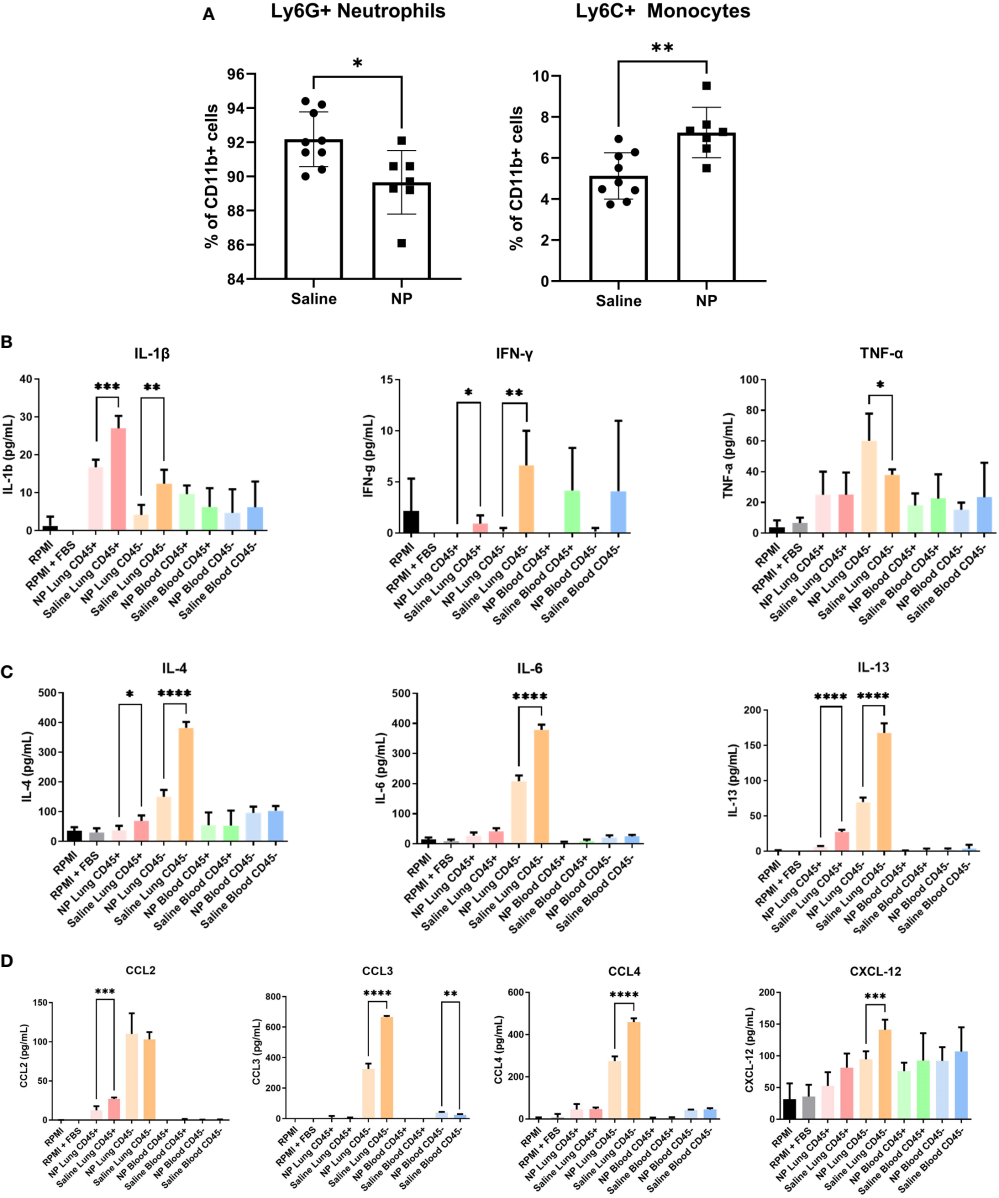
*In vivo* administration of NPs modulates immune cell composition and secretome. **(A)** Flow cytometry quantification of Ly6G+ neutrophils and Ly6C+ monocytes in peripheral blood of 4T1-bearing mice that received NPs (n=7) or saline (n=9). Ratios are calculated as % of CD11b+ myeloid cells. CD45+/- cell fractions were magnetically sorted form peripheral blood and lungs, and cultured for 16h *in vitro*. Secretome was analyzed with ELISA (n=5 mice per group) for cytokines associated with **(B)** Th1 and **(C)** Th2 responses, and **(D)** chemokines. Two-tailed unpaired t-tests assuming unequal variance were performed for single comparisons between two conditions, * p ≤ 0.05, ** p ≤ 0.01, *** p ≤ 0.001, **** p ≤ 0.0001.

Since immune cells at the pulmonary metastatic niche are recruited from circulation, we next investigated how NPs influence the immune cell composition of the peripheral blood (41). Mice injected with NPs had a significantly higher proportion of monocytes and a lower proportion of neutrophils in their peripheral blood (**Figure 4A**). However, the absolute magnitude of change was small, with a 2.1% increase in monocytes and 2.5% decrease in neutrophils. We thus sought to investigate whether NPs influenced the secretome of cells in the blood and at the lungs. Cells from the lungs and blood were isolated into CD45+ and CD45- fractions and cultured for 18 hours, and the resulting supernatant was analyzed by ELISA. In the lung, NP administration broadly decreased the secretion of cytokines and chemokines in both the CD45+ and CD45- fractions (**Figures 4B–D**). CD45+ immune cells in the lung demonstrated significant reductions in the secretion of both Th1 (IL1β, IFNγ) and Th2 (IL-4, IL-13) cytokines. CD45- lung cells also displayed reduced secretion of Th1 and Th2 cytokines (IL-1β, IL-4, IL-6, IL-13). NP treatment also reduced the secretion of chemokines by both CD45+ (CCL2) and CD45- cells (CCL3, CCL4) in the lung. Interestingly, while NP administration reduced the secretion of most tested cytokines and chemokines, NPs conversely resulted in a 1.6-fold increase in the secretion of TNFα by CD45- lung cells. NP administration did not significantly alter the secretion of cytokines or chemokines in CD45+ or CD45- cells in the blood, with the exception of a 1.7-fold increase in CCL3 secretion by CD45- cells. Taken together, these data show that nanoparticles alter the ratios of immune cells in circulation and the secretion of cytokines and chemokines, reducing pathological inflammation in the lung.

### Efficacy of nanoparticles in resection model and their abrogated efficacy in a T-cell-deficient model

**Figure 5:**
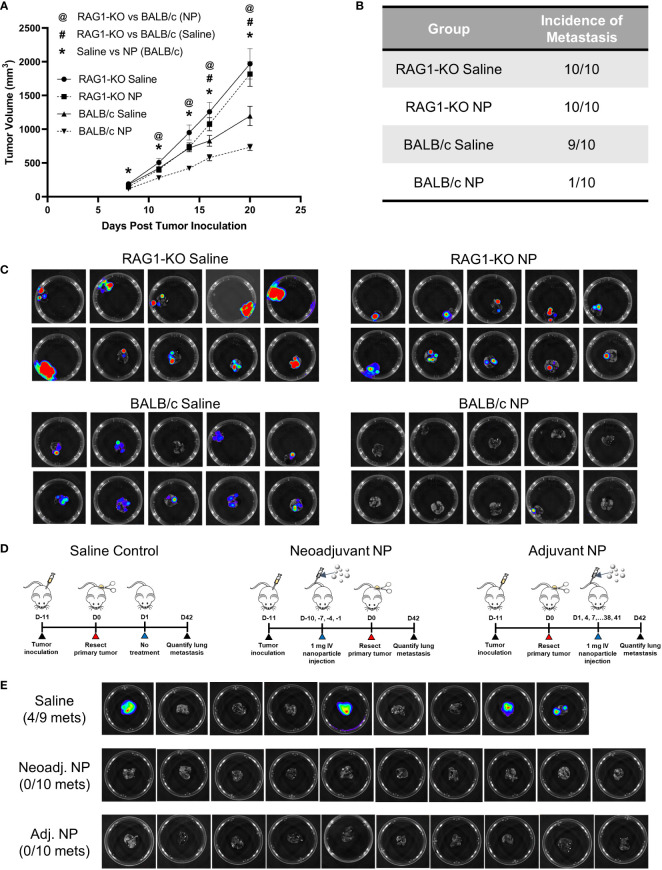
The NP-mediated clearance of metastatic tumors *in vivo* is dependent on functional T-cells. **(A)** Longitudinal PT growth in 4T1-bearing BALB/c or RAG1-KO mice receiving saline or NPs (n=10 per group). **(B)** Proportion of BALB/c versus RAG1-KO mice with pulmonary metastases at D21 post-tumor inoculation and **(C)** BLI of the lungs. **(D)** Schematic of PT resection model and treatment. NPs were administered either before (neoadjuvant) or after (adjuvant) PT resection. **(E)** Lung BLI of BALB/c mice receiving NPs or saline (n=9-10 per group) in PT resection study. Two-tailed unpaired t-tests assuming unequal variance were performed for single comparisons between two conditions. Statistically significant differences in PT growth (p < 0.05): RAG1-KO NP vs BALB/c NP (@), RAG1-KO saline vs BALB/c saline (#), and BALB/c NP vs BALB/c saline (*).

As NP treatment led to an induction of signaling pathways associated with adaptive immunity, we investigated the role of T-cells in the clearance of metastases using a T-cell-deficient mouse model. RAG1-KO and BALB/c mice were inoculated with orthotopic 4T1 tumors and received either NP treatment or saline control. Longitudinal primary tumor (PT) volumes were recorded for the cohort of mice (**Figure 5A**). As observed previously, NPs significantly reduced PT growth in BALB/c mice. Conversely, no statistical difference was observed between the PT volumes of RAG1-KO mice receiving NPs and those receiving saline, across all time points. These data demonstrate that, without mature T-cells, NPs did not reduce PT growth. Next, the impact of T-cells on the NP-mediated inhibition of pulmonary metastasis was investigated by imaging the lungs of tumor-bearing mice. Lungs were collected from NP- or saline-treated BALB/c and RAG1-KO mice 21 days after tumor inoculation. Consistent with our previous studies, metastatic colonization of the lungs was completely inhibited in 9/10 BALB/c mice receiving NPs (**Figures 5B, C**). In contrast, NPs had no effect in the T-cell deficient mice. The entire cohort of RAG1-KO mice developed lung metastases, regardless if NPs or saline was administered (**Figures 5B, C**). Collectively, these data demonstrate that NP-mediated inhibition of lung metastases relies on the presence of a functional adaptive immune response.

Since mature T-cells were necessary for the NP-mediated reduction of pulmonary metastases, we hypothesized that NP administration modulated the immune system to promote tumor cell clearance. We investigated this hypothesis by utilizing a PT resection model, with the goal of studying the impact of NPs on established metastases in the absence of the PT. BALB/c mice were inoculated and split into three cohorts receiving saline control, neoadjuvant nanoparticle treatment (NP administration pre-resection), or adjuvant nanoparticle treatment (NP administration post-resection) (**Figure 5D**). Excitingly, NP administration completely abrogated lung metastases in both the neoadjuvant and adjuvant cohorts, compared to 44% of saline-treated mice developing metastases (**Figure 5E**). The absence of metastases in the adjuvant cohort, in which NPs were delivered after the formation of established metastases, indicates that NP treatment led to the clearance of pulmonary metastases. Taken together, these data indicate that T-cells are necessary for the NP-mediated reduction of pulmonary metastases and that adjuvant nanoparticle treatment can induce the clearance of established metastatic lesions.

## Discussion

In this study, cargo-free immunomodulatory PLG NPs were investigated in the 4T1 murine model of TNBC for therapeutic efficacy in the neoadjuvant and adjuvant setting to manage disease and treat metastasis. Immunotherapies have emerged as a powerful tool for treating localized and metastatic cancers, with their continued development being supported by the nearly 6,300 active clinical trials investigating immunomodulatory agents in cancer that have begun since 2020 (42). However, despite the durable responses in some patients and FDA approvals, the majority of TNBC patients are not responsive to checkpoint inhibition. In the randomized, open-label, phase 3 trial (KEYNOTE-119) of the checkpoint inhibitor pembrolizumab in metastatic TNBC patients, investigators observed a 9.6% objective response rate (43). Interestingly, mounting evidence has demonstrated that checkpoint blockade resistance is driven, in part, by immunosuppressive components of the tumor microenvironment, including myeloid cells (16). This report details an immunomodulatory nanoparticle platform that can reprogram innate immune cells to skew the immunosuppressive microenvironment toward an inflammatory, anti-tumor milieu. These NPs have been investigated with other tumor models (B16F10, MC38, LLC) and are effective at limiting growth of the primary tumor, and this manuscript investigates their use for metastatic disease (44).

We found that primary tumor growth in the 4T1 model of metastatic TNBC was reduced as a result of neoadjuvant NP administration. Clinically, the majority of TNBC patients receive neoadjuvant chemotherapy with the goal of reducing PT volume prior to surgical resection (45). In the KEYNOTE 522 trial, 64.8% of patients receiving a combination of pembrolizumab and chemotherapy demonstrated a pathologic complete response. Notably, this combination of immunotherapy and chemotherapy improved the PCR rate relative to either treatment alone (51.2% for chemotherapy or 21.4% for pembrolizumab, as monotherapies). We have previously reported that cargo-free PLG NPs improved the efficacy of checkpoint inhibition in reducing PT growth, when delivered together as a combination therapy (32). Whereas other nanoparticle approaches deliver pharmaceutic agents, these cargo-free NPs function by a distinct mechanism from either chemotherapy or checkpoint blockade, and modulate immune responses based on the physiochemical properties of the NPs (25, 44). These nanoparticles are made of poly(lactide-co-glycolide), a biodegradable, nontoxic, and FDA-approved polymer already being using in the clinic, and as such, have the great potential to supplement existing therapies and improve disease management when administered as a neoadjuvant therapy.

Adjuvant NP administration, following PT resection in a murine model of TNBC, led to clearance of pulmonary metastases. For many cancer types, metastatic disease marks the stage where treatment no longer has curative intent, and disease progression leads to mortality. While significant advances have been made in the treatment of localized breast cancer, few therapies exist to effectively treat metastasis. As such, breast cancer diagnoses have a 99% five-year survival rate for localized disease, yet survival drops to 29% for metastatic disease (46). The disparity in outcomes between primary and metastatic disease necessitates novel strategies for targeting the microenvironments that support metastatic colonization and the subsequent progression of disease. Immunotherapies have been pursued with the goal of stimulating an anti-tumor immune response to treat metastasis. To investigate the efficacy of immunomodulatory, cargo-free NPs in clearing metastatic lesions, we administered NPs in the adjuvant setting, in which therapy is delivered after surgical resection of the primary tumor. This allows for an intervention to be studied in the clinical context of established metastatic lesions without a PT. Excitingly, we observed that adjuvant NP therapy led to the clearance of pulmonary metastases. These results demonstrate the potential for the cargo-free NP platform as a treatment for metastatic disease in advanced TNBC.

NP administration alters the immune cell composition at the metastatic niche, in part through altered cytokine secretion that impacts recruitment from circulation. In particular, the lungs of NP-treated mice had a marked decrease in the proportion of neutrophils as identified by single cell RNA sequencing, consistent with previous reports (32). Neutrophils suppress effector cell responses in the metastatic niche, and neutrophil depletion has been shown to inhibit metastasis (20, 47). While nanoparticle administration resulted in modest changes in immune cell composition in peripheral blood, we observed large changes in neutrophil accumulation at sites of disease that cannot be explained by systemic changes in composition alone. We found that NP administration drastically reduced the secretion of monocyte and neutrophil-attracting chemokines at the lung, suggesting that nanoparticles induce tissue-specific changes in immune cell recruitment. In particular, the NP-induced downregulation of CCL2 secretion by CD45+ cells and CCL3 by CD45- cells in the lung may disrupt the continued accumulation of immunosuppressive myeloid cells at the metastatic niche. Both CCL2 and CCL3 promote the recruitment and retention of monocytes and monocyte-derived macrophages, while CCL3 also supports the accumulation of neutrophils at metastatic sites (10, 48, 49). CXCL12, which was downregulated in the CD45- lung cells with NP treatment, has also been implicated in the recruitment and retention of neutrophils to the metastatic niche (50). Overall, these findings suggest that NP administration reduces the secretion of inflammatory chemokines and recruitment of metastasis-supporting myeloid cells to the lung, thereby inhibiting the formation of an immunosuppressive metastatic niche.

NP-mediated immunomodulation can disrupt metastasis-promoting immunosuppression and promote an anti-tumor inflammatory environment at metastatic sites. The accumulation of immunosuppressive myeloid cells in peripheral blood and at distant tissues correlates with poor clinical outcomes and immunotherapy resistance (17). Myeloid cells suppress T-cell function in advanced metastases through their secretion of reactive oxygen species and surface expression of markers such as PD-L1 (12, 13, 15). In the lungs of NP-treated mice, inflammatory gene expression pathways and inflammatory myeloid cell subsets were upregulated, in comparison to the lungs of saline-treated mice. Signaling pathways associated with IFNγ and TNFα were significantly upregulated in neutrophils, monocytes, and dendritic cells. IFNγ signaling has been associated with anti-tumor cytotoxicity in neutrophils, as well as maturation of monocytes and DCs, which can lead to the activation of tumoricidal NK and T-cells (38, 39). Similarly, TNFα has been associated with neutrophil cytotoxicity and tumoricidal polarization of monocyte-derived macrophages (40, 51). Notably, while TNFα secretion increased with NP administration, IFNγ secretion decreased, suggesting that the upregulation of genes associated with IFNγ signaling may be a direct result of NP internalization. This connection is further supported by the presence of neutrophil and monocyte subpopulations that highly express IFNγ-associated genes in the lungs of nanoparticle-treated mice.

T-cell activity was critical to the NP-induced clearance of metastatic tumor cells. T-cell immunity is a vital component of anti-tumor immune surveillance, and cancer-induced T-cell dysfunction is a major mechanism of immune escape (52). The infiltration of immunosuppressive myeloid cells contributes to T-cell anergy and the recruitment of regulatory T-cells, and previous therapeutic strategies targeting immunosuppressive myeloid cells have demonstrated success in enhancing anti-tumor T-cell activity (53). The delivery of NPs following surgical resection, at a time in which metastases had already seeded the lungs, abrogated metastatic colonization, suggesting that NPs reprogram the metastatic niche to promote the active clearance of metastatic tumor cells. As NPs showed efficacy in immunocompetent mice, but were ineffective in T-cell-deficient mice, this clearance is likely driven by T-cells. While the induction of inflammatory gene expression programs in neutrophils may induce anti-tumor neutrophil cytotoxicity, the lack of response in T-cell-deficient mice demonstrates that tumor clearance by innate immune cells alone is insufficient, and that T-cells are essential to the NP-mediated reduction of pulmonary metastases (54). Interestingly, NP administration significantly increased DC activation and the proportion of Th1/Th2 CD4+ T cells and cytotoxic CD8+ T cells. While we could not distinguish between Th1 and Th2 CD4+ T cells in our data, polymeric nanoparticles have previously been reported to induce Th1 phenotypes, motivating further study on how cargo-free nanoparticles stimulate anti-cancer adaptive immune responses (55, 56).

In conclusion, we report that cargo-free PLG NPs augment PT resection by abrogating distant recurrence when administered either as a neoadjuvant or adjuvant therapy. NPs modulated the immune microenvironment of the lungs, a metastatic site in TNBC, skewing suppressive immune cells toward inflammatory, anti-tumor phenotypes. NP administration enhanced T-cell-mediated tumor cell clearance, and their efficacy was nullified in a T-cell-deficient model. These promising results demonstrate the utility of cargo-free nanoparticles as a novel, immunomodulatory neoadjuvant/adjuvant therapy to mitigate recurrence during the management of advanced cancers.

## Data availability statement

The sequencing data presented in the study are deposited to the NCBI GEO repository under accession number GSE217900.

## Ethics statement

The animal study was reviewed and approved by University of Michigan Institutional Animal Care & Use Committee.

## Author contributions

RR and JM primarily conceptualized the experiments included in this manuscript with input from TM, AE, and LS. Experiments were primarily performed by RR, JM, and YZ with additional experimental and analysis support from SO, JW, MZ, SK, QX, and JP. The paper was drafted by RR and JM with input from SO, JW, MZ, SN, TM, AE, JJ, and LS. All authors contributed to the article and approved the submitted version.

## Funding

Funding for this research was provided by COUR Pharmaceutical Development Co, Inc., and the National Institutes of Health under award numbers 1R01AI148076, 1R01AI155678, 1R01CA243916, and R01CA214384. Transcriptomic analyses were supported by the National Cancer Institutes of Health under Award Number P30CA046592 by use of the following Cancer Center Shared Resource: Single Cell and Spatial Analysis Shared Resource.

## Acknowledgments

We would like to acknowledge the University of Michigan Rogel Cancer Center Immunology Core for assistance with cytokine measurements and the Advanced Genomics Core at the University of Michigan for library preparation and next-generation sequencing of transcriptomic samples.

## Conflict of interest

Authors SK, QX, JP, TM, and AE are employees of COUR Pharmaceutical Development Co, Inc., and author LS consults and has financial interests in COUR Pharmaceutical Development, Inc.

These studies were funded, in part, by COUR Pharmaceutical Development Co, Inc., which holds the intellectual property for nanoparticles studied in this manuscript.

The remaining authors declare that the research was conducted in the absence of any commercial or financial relationships that could be construed as a potential conflict of interest.

## Publisher’s note

All claims expressed in this article are solely those of the authors and do not necessarily represent those of their affiliated organizations, or those of the publisher, the editors and the reviewers. Any product that may be evaluated in this article, or claim that may be made by its manufacturer, is not guaranteed or endorsed by the publisher.

## Supplementary material

Supplementary Figure 1 Material characterization of ONP-302 nanoparticles. **(A)** Representative scanning electron microscope (SEM) image of NPs. **(B)** Quantification of NP size (nm), polydispersity index (PDI), and zeta potential (mV) with dynamic light scattering (DLS).

Supplementary Figure 2 NP administration does not alter the ratio of CTCs to white blood cells in 4T1 tumor-bearing mice (n=6 per group). **(A)** Quantification of the ratio of CTCs to white blood cells (WBCs). Cell quantities normalized to blood volume. **(B)** Representative images of cells isolated from peripheral blood. Blue – DAPI, Green – CD45+, Red – tdTomato.

Supplementary Figure 3 Gene expression profiles of lung-derived cell populations by single cell RNA sequencing. **(A)** Marker genes used to identify immune cell clusters. Gene expression of **(B)** neutrophils, **(C)** dendritic cells, and **(D)** monocytes used to identify subpopulations.

Supplementary Figure 4 NP administration upregulates activated phenotypes in T cells. **(A)** Histogram (left) and UMAP plot (right) of single cells at the lungs of NP or saline-treated mice (n=4 per group) identified as T cells in . **(B)** Marker genes used to identify T cell subsets.

Supplementary Table 1 List of genes used to assign identities to single cell clusters in .

Supplementary Table 2 List of genes used to classify **(A)** neutrophil, **(B)** monocyte, and **(C)** dendritic cell subsets in .
