# Supplementary figures and images for: Myeloid cell reprogramming alleviates immunosuppression and promotes clearance of metastatic lesions
Source: Front Oncol. 2022 Nov 21;12:1039993. doi: 10.3389/fonc.2022.1039993 (PMC9720131; doi:10.3389/fonc.2022.1039993)

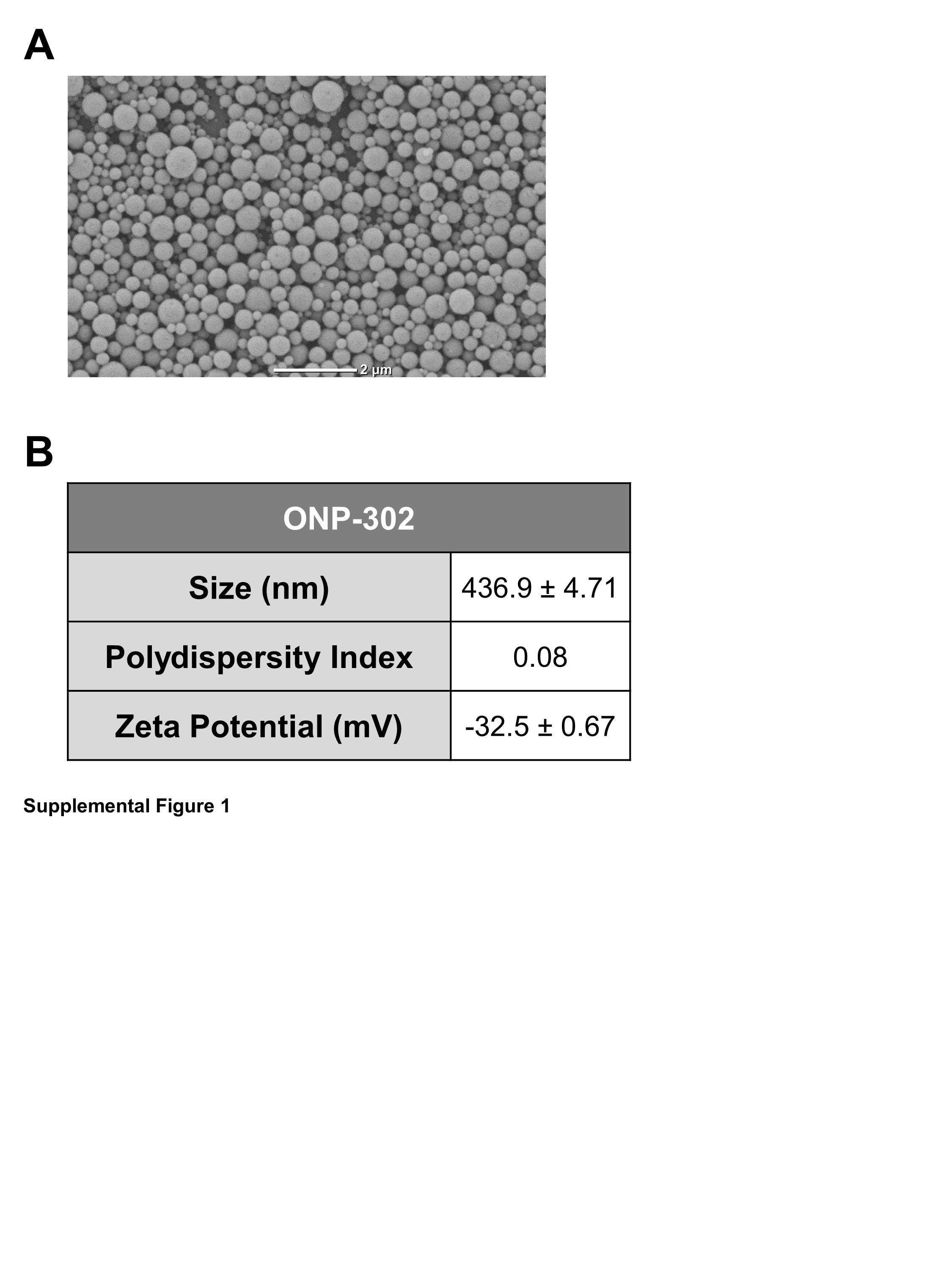

Supplement: Supplementary Figure 1 — Material characterization of ONP-302 nanoparticles. (A) Representative scanning electron microscope (SEM) image of NPs. (B) Quantification of NP size (nm), polydispersity index (PDI), and zeta potential (mV) with dynamic light scattering (DLS). [file Image_1.tif]

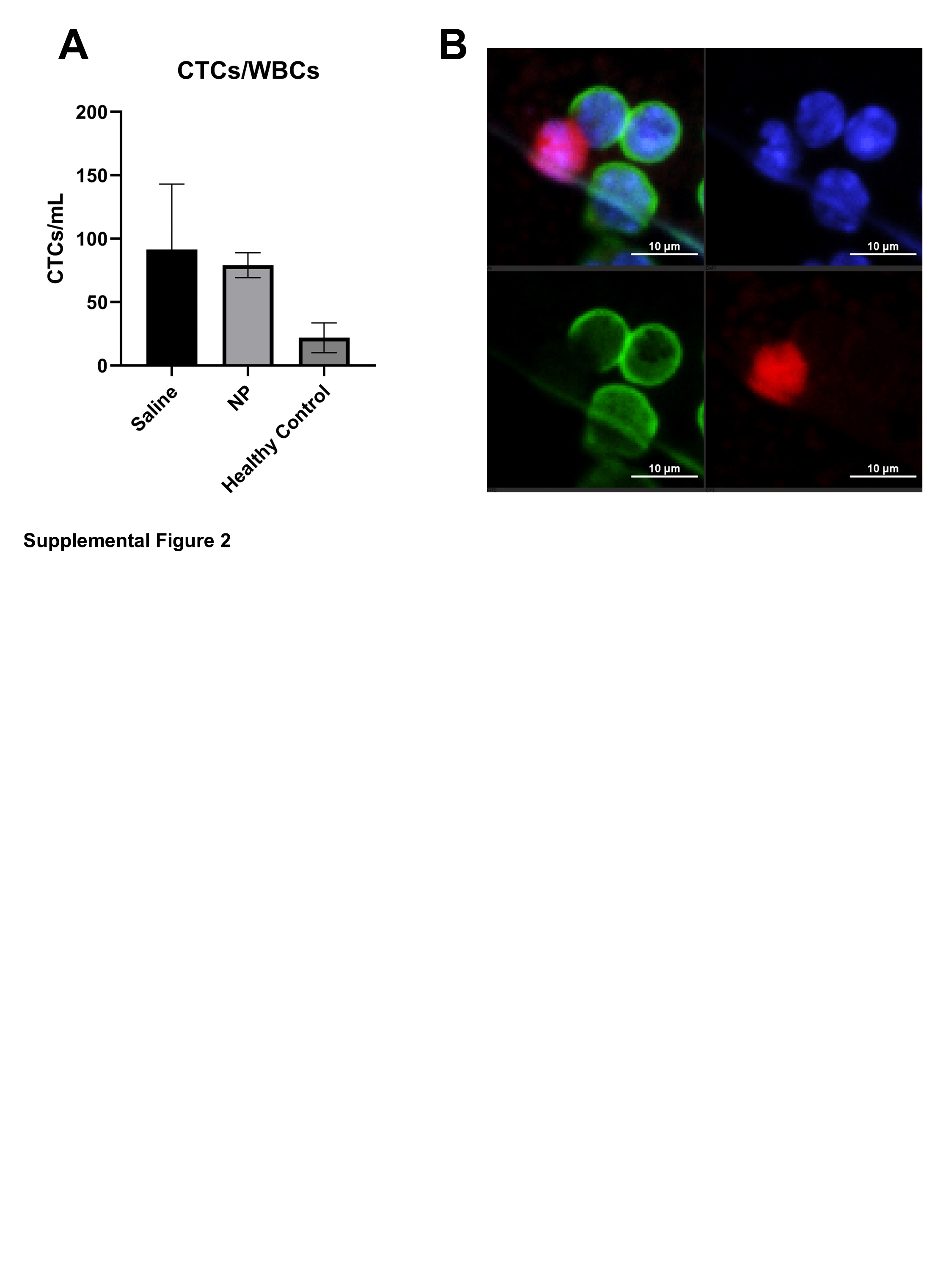

Supplement: Supplementary Figure 2 — NP administration does not alter the ratio of CTCs to white blood cells in 4T1 tumor-bearing mice (n=6 per group). (A) Quantification of the ratio of CTCs to white blood cells (WBCs). Cell quantities normalized to blood volume. (B) Representative images of cells isolated from peripheral blood. Blue – DAPI, Green – CD45+, Red – tdTomato. [file Image_2.tif]

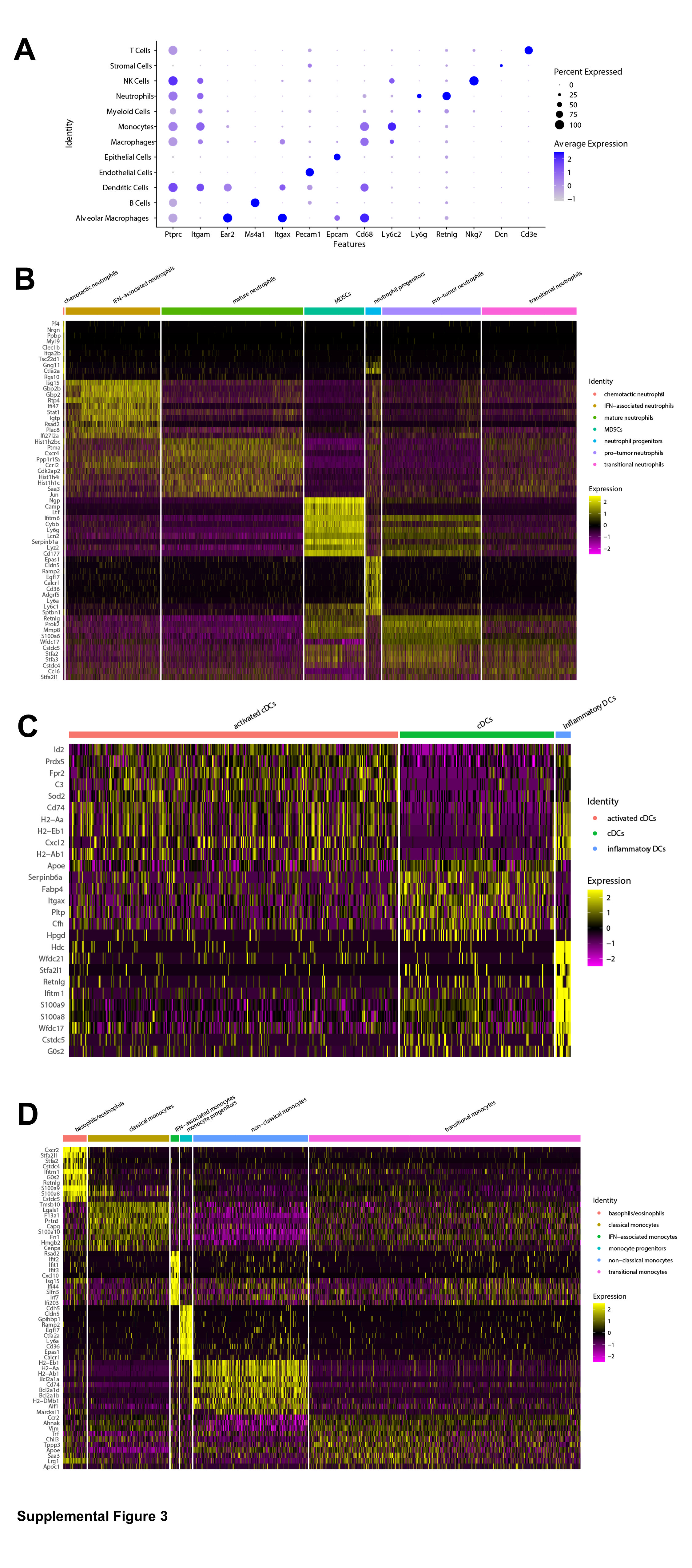

Supplement: Supplementary Figure 3 — Gene expression profiles of lung-derived cell populations by single cell RNA sequencing. (A) Marker genes used to identify immune cell clusters. Gene expression of (B) neutrophils, (C) dendritic cells, and (D) monocytes used to identify subpopulations. [file Image_3.tif]

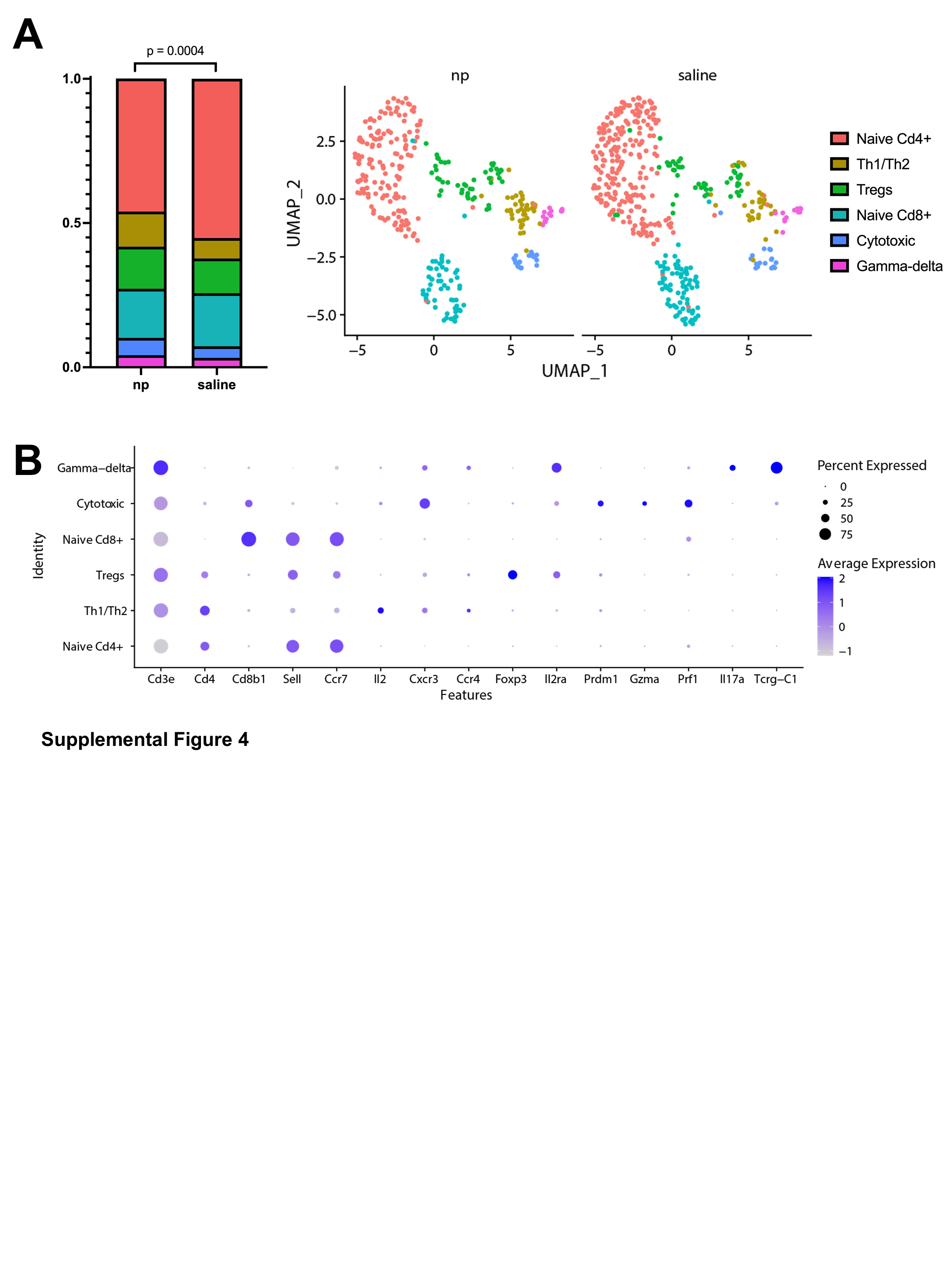

Supplement: Supplementary Figure 4 — NP administration upregulates activated phenotypes in T cells. (A) Histogram (left) and UMAP plot (right) of single cells at the lungs of NP or saline-treated mice (n=4 per group) identified as T cells in . (B) Marker genes used to identify T cell subsets. [file Image_4.tif]
